# Commentary: Identification of IFN-Induced Transmembrane Protein 1 With Prognostic Value in Pancreatic Cancer Using Network Module-Based Analysis

**DOI:** 10.3389/fonc.2021.707516

**Published:** 2021-07-20

**Authors:** Xinxiao Li, Renba Liang, Liu Yang

**Affiliations:** ^1^ Department of Oncology, Affiliated Hospital of Guangdong Medical University, Zhanjiang, China; ^2^ Department of Radiation Oncology, National Cancer Center/National Clinical Research Center for Cancer/Cancer Hospital & Shenzhen Hospital, Chinese Academy of Medical Sciences and Peking Union Medical College, Shenzhen, China

**Keywords:** IFITM1, cancer, treatment, progress, target

The human interferon (IFN)-induced transmembrane protein 1 (IFITM1), also called Leu13 or CD225, is a 17-kDa cell-surface membrane protein in the IFN-stimulated genes (ISGs) protein family (1). IFITM1 was initially identified as a leukocyte membrane surface antigen involving in signal transduction in lymphocytes, such as antiproliferative and homotypic adhesion signaling (2, 3). Additionally, IFITM1 was regarded as a modulator of immunity and antiviral activity (1). Recently, with great interest we read a study “Identification of IFN-Induced Transmembrane Protein 1 With Prognostic Value in Pancreatic Cancer Using Network Module-Based Analysis” in Frontiers in Oncology (4), revealing that the expression level of IFITM1 was increased in pancreatic cancer. Patients with IFITM1 overexpression had poor survival, and IFITM1 was one of the independent prognostic factors for overall survival. Moreover, down-regulation of IFITM1 significantly suppressed the tumorigenicity of pancreatic cancer cells (4). In addition, increasing evidence have demonstrated that IFITM1 was also upregulated in numerous tumor tissues as well as cancer cell lines, such as colorectal cancer (5), gastroesophageal adenocarcinoma (6), gastric cancer (7, 8), hepatocellular carcinoma (9), lung cancer (10), breast cancer (11, 12), head and neck cancer (13, 14), gallbladder carcinoma (15), ovarian cancer (16), glioma (17), and nasopharyngeal carcinoma (18). Overexpression of IFITM1 enhanced cell proliferation, invasion, metastasis, angiogenesis, and therapeutic resistance, including endocrine therapy, chemotherapy, and radiotherapy resistance of tumors. Mechanically, IFITM1 exerted cancer-promoting effects through regulating serval pathways, including EGFR/SOX2 (10), JAK/STAT (11), and CAV-1 (19). These findings suggest that pharmacological or genetic inhibition of IFITM1 maybe a potential and novel therapeutic approach for tumors.

## Author Contributions

Study concept and design, XL. Data analysis, methodology, drafting manuscript, RL. Review and editing, supervision, LY. All authors contributed to the article and approved the submitted version.

## Conflict of Interest

The authors declare that the research was conducted in the absence of any commercial or financial relationships that could be construed as a potential conflict of interest.
